# Transparency and sponsored content in social media: Considerations for dermatologists

**DOI:** 10.1016/j.jdin.2024.08.004

**Published:** 2024-09-24

**Authors:** Cassidy Nguyen, Fatima Khan, Lia E. Gracey

**Affiliations:** Division of Dermatology, The University of Texas at Austin, Dell Medical School, Austin, Texas

**Keywords:** dermatologist, ethics, Instagram, partnership, product branding, social media, sponsorship, Tiktok

*To the Editor:* With the growing international influence of social media within dermatology, we sought to examine the ethical considerations and impact of skin-related advice and brand sponsorships from dermatologists and self-proclaimed “skin care experts.” We have read Trepanowski’s paper on “Social media dermatologic advice: dermatology without dermatologists” and agree that compromise to patient health is a significant concern given varied dermatologic advice on social media.^1^ While dermatologists can disperse more accurate health information on social media, we would like to highlight the ethical implications of dermatologists’ role in social media and paid sponsorship.

Dermatologists are in a unique position given they provide recommendations on not only prescription management but also various over-the-counter skin products, leading to an overlap with the cosmetics industry. While offering guidance amidst the overwhelming array of skincare products can be helpful, the introduction of sponsored content on social media blurs the line between genuine recommendation and commercial interest.

At the heart of this issue lies the principle of beneficence, or the duty to act in the best interests of the patient. The Hippocratic oath and the physician’s obligation to “do no harm” apply to all physicians, who have professional and ethical obligations to recommend treatments that are safe and supported by scientific evidence. However, Hippocrates may not have imagined a world of sponsored content and the dissemination of medical advice via social media. Unlike peer-reviewed research, in which authors must disclose their conflicts of interest, no established guidelines exist for social media apart from the Federal Trade Commission guidelines for endorsements, which are nebulous themselves.^2^ While the AMA Code of Medical Ethics comments on the use of professionalism in social media, there is no mention of paid sponsorships and how this plays into the principle of nonmaleficence.^3^ Thus, when it comes to conflicts of interest, physicians should never put their financial interests above the welfare of patients.

A recent study analyzing dermatology’s presence on Tiktok (with over 1 billion active users) demonstrated that while less than half of dermatologists had sponsored posts for skincare brand recommendations, those who did participate in brand endorsements had more followers.^4^ Similarly, on Instagram (with over 2 billion active users), 45% of dermatology residents/fellows and 30% of attendings who posted content participated in sponsored posts, though not all were directly related to dermatology.^5^

We call for transparency on social media platforms to clearly signify sponsored content. Board-certified dermatologists should have a voice on social media, and if they engage in sponsored content, it should be disclosed if any monetary benefit is received. We call for dermatologists who post product recommendations on social media to disclose to followers: (1) Their credentials (MD, PhD, DO, and board certification); (2) If they are being paid to endorse the product; and (3) Whether the product is backed by validated scientific studies in order to maintain trust between physicians and the public. Overall, when done with careful thought, dermatologists may provide a positive impact in helping patients navigate this continuously evolving digital age.

## Conflicts of interest

Dr Gracey is a member of the American Academy of Dermatology (AAD) Teledermatology committee (uncompensated) and the AAD Image Collection Workgroup (honoraria), neither of which were directly relevant to this publication. Authors Nguyen and Khan have no conflicts of interest to declare.
